# The Power of Radio to Promote Health and Resilience in Natural Disasters: A Review

**DOI:** 10.3390/ijerph16142526

**Published:** 2019-07-15

**Authors:** Karin Hugelius, Mike Adams, Eila Romo-Murphy

**Affiliations:** 1 School of Health Sciences, Örebro University, 70182 Örebro, Sweden; 2 First Response Radio, Frome BA11 2SX, UK; 3 Health Communication Resources (UK), Worthing BN13 1AA, UK

**Keywords:** community resilience, crises communication, disaster, disaster response, humanitarian radio, mental health

## Abstract

Humanitarian radio has been used in humanitarian aid efforts and after natural disasters over the last 15 years. However, the effects have barely been evaluated, and there are few scientific reports on the impact of radio as a disaster health response intervention. Therefore, this study aimed to provide an overview of the use and impact of humanitarian radio in natural disasters from a health perspective. A literature review of 13 scientific papers and grey literature resources was conducted. The results show that humanitarian radio could be used to promote both physical and psychosocial wellbeing by providing health-related information, advice and psychosocial support in natural disasters. Community resilience can be enhanced by the promotion of community engagement and can strengthen self-efficacy and community efficacy. Radio also has the potential to cost-effectively reach a large number of affected people in areas with severely damaged infrastructure. Radio could, therefore, contribute to health recovery and wellbeing from both individual and community perspectives. As such, health professionals; crises communication professionals, including radio journalists; and disaster-managing stakeholders should be prepared and trained to use humanitarian radio as an integrated part of the disaster health response in natural disasters.

## 1. Introduction

In 2017, 335 natural disasters affected over 95.6 million people. These disasters mostly occurred in Asia in the form of storms or floods, but they also affected other parts of the world as earthquakes, storms and wildfires, which collectively caused deaths, injuries and human desolation [1]. Natural disasters usually cause physical injuries and associated complications [2,3] and worsen chronic conditions [4]. Damage to infrastructure and medical facilities is typical after some types of natural disasters and can impair the ability to manage both acute and chronic health conditions [5]. The mental health effects of disasters include a range of problems, some of which are psychopathologic [4,6,7]. Disturbances in social relations, economic consequences and temporary or definitive displacements also cause distress in disaster-affected populations [7,8,9]. Therefore, health effects after natural disasters must be considered as physical, psychological and social. 

Today, crisis communication is considered to be an integrated part of disaster health response [10]. Access to reliable information in a language that is easily understood and culturally appropriate is part of the Core Humanitarian Standard [11]. Establishing communication and access to reliable information can facilitate health recovery and reduce mortality after disasters [12,13]. From a historical perspective, public radio has been used worldwide as a communication method in emergencies and disaster situations, mainly for communication on disaster preparedness, risk awareness or risk reduction [10,14]. There are many terms to describe the use of radio in humanitarian contexts, including ‘disaster radio’, ‘emergency radio’, ‘beneficiary radio’, ‘radio in a suitcase’, and ‘radio in a box’. In this paper, ‘humanitarian radio’ will be used, meaning a specific radio station that broadcasts disaster-related information, specifically aiming to promote recovery among the listeners by either temporary technical solutions or ordinary means [15]. Local emergency management authorities (LEMA), non-governmental organizations (NGOs), United Nations (UN) agencies, and the Red Cross and Red Crescent Movement are examples of stakeholders that have been involved in providing humanitarian radio in emergencies during the last several decades [14]. However, evaluations and studies on the use of radio, including the health effects of the use of radio, as a disaster response intervention have been very limited, and no review papers are currently available. Therefore, the purpose of this paper is to provide an overview of the use and impact of humanitarian radio as a disaster health response intervention with a particular focus on natural disasters. 

## 2. Materials and Methods

A literature review, which was inspired by the integrative review method [16] and based on research studies and grey literature, was conducted. This kind of review summarizes past empirical and theoretical literature to achieve a comprehensive understanding of a specific problem or phenomenon [16]. 

### 2.1. Search Strategy 

As a first step, a structured search of the Medline database and Web of Science was made, focusing on original research papers that aimed to describe the use or impact of radio from a health perspective during or in the aftermath (the first three months) of a natural disaster. Papers published in English between 1998 to 2018 were included in the present review. Papers describing the use or impact of Very High Frequency (VHF) radio communication and papers focused on disaster preparedness or prevention, including early warning systems, were excluded. Papers describing capacity-building initiatives in complex humanitarian emergencies or conflicts were also excluded. Keywords were combined in order to identify the available sources that met the inclusion criteria for this review (See Table 1). Next, a grey literature search was conducted using the same inclusion criteria. Manual searches of the website of the Communicating with Disaster Affected Communities (CDAC) Network and the websites of all CDAC member agencies [17] were made. As a third step, a manual search of the reference lists of the identified papers was conducted. No further sources to include in the review were identified by this manual search. 

**Figure 1:**
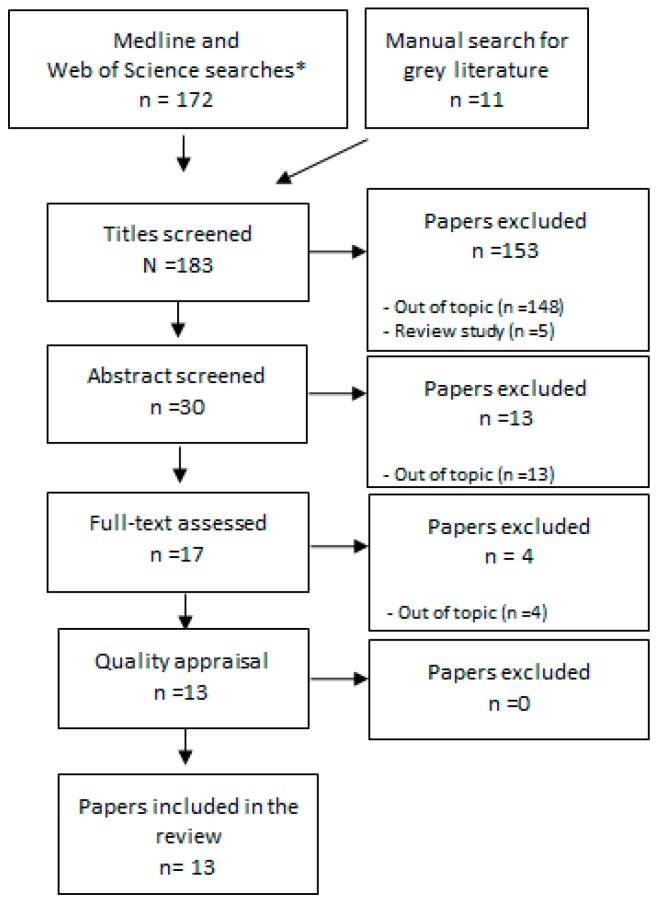
Overview of the process to identify papers included in the review. * Search valid as of 4 March 2019.

The search for studies in Medline and Web of Science yielded 172 papers. The grey literature search yielded 11 reports or papers. A reading of the titles reduced the number of possibly relevant papers to 30. The most common reasons for being excluded at this phase was reporting via VHF radios or amateur radio instead of radio broadcasting or referring to ‘radio’ as in radio nuclear studies. Also, papers describing the use of radio in humanitarian contexts other than natural disasters were common. In total, 17 papers and reports remained after the above exclusions; each paper was read in its entirety, and 13 papers or reports were selected for the analysis stage. The search strategy and results are presented in Figure 1.

### 2.2. Quality Appraisal

An original integrative review used quality scoring in order to conduct a quality appraisal [16]. Due to the inclusion of grey literature, this review study used a simplified quality appraisal, grading the quality of the primary sources as adequate or not. If a source provided brief descriptions of its methodology, sample, sample size, instruments and analysis used, it was assessed to be ‘adequate’. If a source was graded as ‘not adequate’, no information on the methods used to gather information or synthesize the conclusions was recorded. One of the authors of the present review was a co-author of several of the included papers. Therefore, an external evaluator was asked to grade those papers as adequate or not adequate, based on the above-mentioned criteria. All papers identified as relevant for the review were found to be adequate and could, therefore, be included in the analysis process.

### 2.3. Analysis

After identifying the papers and reports that met the inclusion criteria, all documents were read several times in their entireties, and the data relevant to the purpose of this review were identified, extracted and reduced. Among the included research studies, only data from the results and conclusion sections were used in the present analysis. For the grey literature sources, all relevant data considered as a result or a conclusion was included in the analysis. Then, the extracted data were sorted into groups and clusters related to the aim of the study (humanitarian radio as a disaster health response intervention), and the reviewers compared and contrasted the information before summarizing the analysis into an integrated synthesis [16]. 

## 3. Results 

The research studies and reports used in this study were drawn from 13 papers, covering the use or impact of humanitarian radio in several large-scale natural disasters (see Table 2). 

The results will be discussed below in sections covering the practical use of radio, physical health promotion, psychosocial health promotion and the promotion of community resilience. 

### 3.1. The Use of Radio in Natural Disasters

Radio has been used in the immediate response phase of many kinds of natural disasters and with a range of stakeholders, dispatching messages, information or advice to affected communities [18,19,20,21,22]. One practical example of a temporary solution to provide humanitarian radio in an emergency is the ‘radio in a suitcase’ concept. This device contains all the technical means necessary to broadcast radio, such as a mixer, an MP3 player, a voice recorder, a computer, a microphone, a 600-W FM transmitter and an antenna packed into two 20-kilogram suitcases and gator flight cases [23]. Radio is advantageous in that it can reach a large number of people with limited technical resources and at a low cost [19]. When no electricity is available—and, therefore, mobile phones, televisions and ordinary media channels are also not available—solar or wind-up radios can be easily distributed and used sustainably without any specific skills [19,22,23]. Also, radio can be broadcast on loudspeakers [22,24,25]. 

The providers of humanitarian radio were most often NGOs (based on request from the affected country) in all included studies except three, where ordinary public service radio was used [26,27,28]. Several stakeholders, including local authorities [21,22,26,28,29], UN agencies [22,24], NGOs [18,19,24] and private companies [22], used humanitarian radio as a means of transmitting their information to the affected community. Among these stakeholders, actors from the health sector [18,19,21,22,25,27,29], water and sanitation [22,28], protection [19,24] and education [19] were represented. 

To illustrate the use of radio as a natural disaster response intervention, disaster response operations by the NGO, First Response Radio (FRR) [23], are presented in Table 3. The exact reach of the broadcasts and information on how many people in the affected area actually listened to the radio were hard to estimate and were only reported in one study [25]. In a self-selected internet-based survey of 442 survivors from the Haiyan typhoon (The Philippines), 67% (*n* = 296) stated that they listened to disaster radio at some time during the first months after the disaster event. The reasons for listening to the radio were to obtain practical advice (71%, *n* = 214), for company (57%, *n* = 169), to obtain information on available services (50%, *n* = 149), to obtain information on the wellbeing of relatives and friends (33%, *n* = 98), to take a break (30%, *n* = 87) and to obtain general information (22%, *n* = 65) [25]. 

### 3.2. How Did Radio Promote Physical Health? 

Radio has been used in many natural disasters to broadcast health-related information. By providing practical advice and stimulating self-care for both physical and mental health problems, wellbeing among survivors can be supported. The health-related information broadcasts after the Haiyan typhoon [22] showed that radio was used to advise survivors on the availability and distribution of non-food items, e.g., blankets and hygiene kits, and behaviors to stay healthy, e.g., advice on how to boil water and detect signs of diseases. Also, radio broadcasts provided information on immunization programs, the policy of a general health insurance company, the management of dead bodies and how to avoid and recognize specific health threats, such as dengue fever and leptospirosis [22]. In addition, radio was used to inform people on where to find medical services, such as what hospitals were open and whether temporary medical units, such as field hospitals, were available [22]. In the Ebola crises in Western Africa, radio was used to inform listeners about the signs of Ebola, personal protection and preventive measures [29]. All of this information aimed to promote physical health [19,22,28]. 

A multiple linear regression analysis on variables influencing perceived health among survivors 30 months after the Haiyan typhoon indicated that listening to the provided humanitarian radio after the typhoon positively influenced the general health of the survivors [30].

### 3.3. How Did Radio Promote Psychosocial Wellbeing? 

Radio communications have been used in many natural disasters to transmit information, clarify situations and inform people about available support [18,20,22,24]. As such, radio can reduce stress and contribute to feelings of control and clarity among the listeners, which are important for the recovery process [31]. Psychoeducation was broadcasted after the Haiyan typhoon, Hurricane Katrina and the Ebola epidemic in Western Africa [22,26,31], covering information about stress reactions and advice on how to cope with them [22,26,31]. Radio contributed to an increased sense of normality, providing moments of rest from the straining survival and recovery process among those affected [18,31]. After Hurricane Katrina, radio was used to inform people about post-traumatic stress, and an increased awareness among listeners of such problems and available help was measured [27]. Following the Haiyan typhoon, radio broadcasts of information and music were both found to contribute to feelings of hope and connectedness among the survivors [31]. Listening to pop music and familiar ‘happy songs’ positively influenced the wellbeing of the listeners. During night-time, calmer, but still recognizable, music, such as popular ballads and traditional music, was played to instill a feeling of serenity [31]. 

### 3.4. How Did Radio Promote Community Resilience? 

In countries where radio is a trusted source of information or entertainment, humanitarian radio has been shown to be of importance as a source of both information and support after a natural disaster, as well as in situations where all other means of communication were lost [18,21]. Radio promoted both a sense of self-efficacy and community efficacy by inviting people to contribute to the recovery process and promoting community engagement in several natural disasters [24,31]. In a case study from Nepal, listening to interviews with people from the affected communities and representatives from the authorities, religious entities and relief organizations promoted a sense of unity among the listeners, and was found also to stimulate community participation in the recovery process [24]. During the Ebola outbreak in Western Africa, encouraging people to call into programs and ask questions and involving people from the affected communities in the production of radio broadcasts was found to stimulate community engagement [21]. Furthermore, using positive language when broadcasting messages promoted a sense of cooperative resilience among the affected population [29]. Well-known and trusted journalists or local leaders, when used as mediators, increased the adherence to the radio messages during the Ebola epidemic [29]. Radio was used as a child-friendly health promotion intervention, by producing a radio-based education program when schools were closed during the Ebola epidemic in Sierra Leone. This gave children a voice and was found to be an effective and low-cost solution to promote normality and involve children and youths in community engagement activities [19]. 

## 4. Discussion

Promoting the health and wellbeing of a large number of people in distress in environments without functional infrastructure, such as electricity, roads and ordinary means of communication, is a great challenge. Such circumstances are the reality in most natural disasters. This review has demonstrated how disaster radio may be used to promote health during and in the aftermath of such a situation. 

By actively advising people on where to turn for medical services and promoting self-care advice, available medical resources may be more effectively used. Furthermore, proactively raising public awareness on how to reduce health problems might ease the burden on medical services. Radio campaigns have been proven to create substantial changes in health-seeking behaviors and reduce mortality in non-emergency contexts in Burkina Faso [32]. There is no reason to doubt that humanitarian radio used in natural disasters would not have the same positive effects. However, since randomized control studies are nearly impossible to conduct in the immediate aftermath of a natural disaster [33], such conclusions cannot be drawn from this review and may not be possible in the future. 

From a historical perspective, awareness of the mental health impacts of disasters and the demand for psychosocial support after such events have increased [34]. Information is also an essential part of the mental health response [35]. Psychosocial support in the emergency phase should rely on five evidence-based principles—that is, everything possible should be done to promote (1) a sense of safety, (2) calmness, (3) a sense of self-efficacy and community efficacy, (4) connectedness and (5) hope [36]. By providing information about what has happened and advising people on how to stay safe, radio can enhance a sense of safety. Information itself is a well-known factor contributing to the creation of feelings of control and clarity, and both of these are important for the recovery process [36,37]. Promotion of a sense of self-efficacy, as well as community efficacy, has also been found to be beneficial for people in distress [36]. Furthermore, the perception that one is capable of managing the demands related to a disaster and active engagement are associated with a positive recovery process [38]. Radio contributes to all this by providing information, reporting on the authorities’ abilities to manage the situation and encouraging self-supporting activities and self-efficacy. Some of the affected people in a natural disaster will be able to maintain their psychosocial wellbeing if their access to social networks is ensured [35], and social support has been identified as the most important factor in promoting recovery after traumatic events [39,40]. When a person lacks their ordinary social support from family or close friends, hearing another voice on the radio has been shown to serve as an alternative and reduce feelings of isolation and loneliness. Radio can also provide practical advice on how to re-establish contact with loved ones. However, it is important to note that, most often, radio must be complemented with other response activities in order to re-establish social networks, such as providing internet and mobile phone networks or offering to locate missing family members. 

When promoting hope, large-scale, community-based interventions may be more effective than individual interventions [36]. This review showed that music played on the radio resonated specific values and meanings for affected people and was found to increase their feelings of hope and trust. The healing health effects of music are well known but, scientifically, not fully understood [41]. This implies an indication for further research on how music broadcasted through radio can be used to promote health and wellbeing in disasters. It should also be noted that humanitarian radio does not only offer one-way communication, but it gives the community a voice and provides a platform for community engagement. This is vital for humanitarian response in general and contributes to sustainable community resilience [42]. 

This review shows that radio has been used and has played an important role in natural disasters, not only in low- or middle-income countries and regions, such as the Philippines and Western Africa, but also in high-income countries, such as the United Kingdom and the United States. Today, the use of mobile applications and social media for information purposes and social contacts has increased and may also be tools that can be used in disasters. However, it should be remembered that these tools require a functioning internet service, and natural disasters often cause severe interruptions in mobile and internet coverage, sometimes for a long time. The studies included in this review all emphasized that radio was available and operational in situations with severely injured infrastructure, when the internet and mobile phones were not functioning. Also, studies suggest that people in emergencies and disasters tend to lean on information sources to which they are accustomed [20], and a large proportion of social media users were under-represented in the research [43]. Therefore, traditional radio still has an important role to play in disasters but may also be complimented by social media, mobile applications and other communication technology. 

It should be noted that there are many disasters where radio has been used as a response intervention, but there are only a few structured evaluations or research studies available. Quick disaster response requires preparation. For humanitarian radio, preparations should not only include training teams and logistical preparations but also legal procedures, such as those needed to gain the necessary permissions to broadcast. Radio broadcasters also need to have an understanding of the difference between ordinary media reporting and humanitarian radio as a disaster response intervention [44]. This includes basic knowledge on disaster management and the response system, human needs and stress reactions. In the same way, health professionals involved in a disaster response need to be aware of the importance of communication and the advantages of using radio as a health promotion tool. In order to enhance the very best practices of using radio as an effective health response intervention, this review has highlighted the obvious need for close cooperation between the local emergency management authorities, the provider of humanitarian radio and health professionals. 

### Limitations

The limited number of scientific papers describing the use or impact of humanitarian radio from a health perspective made it impossible conduct a traditional systematic literature review based only on scientific papers. Mixing scientific published papers and grey literature added much value to this review. Grey literature can provide data not found elsewhere and can make important contributions to the synthesis of knowledge [45], especially in public health research [46]. The findings in this review were based on a limited number of disaster events and an even more limited number of scientific studies. Some of the studies included in the review were written by one of the authors who synthesized this review. However, if handled with objectivity, the inclusion of one´s own papers is not controversial in literature reviews [47], and an external person made the quality appraisal for these studies. All data in this review were based on non-randomized, convenient samples or qualitative studies. However, the lack of randomized controlled studies does not necessarily mean that the intervention is not useful or effective, and an integrative review can contribute to building evidence on use, effects and best practice [16]. In the search stage, many studies were found to report the use and effectiveness of radio as a preparedness or warning mediator. It is, therefore, a bit remarkable that the scientific interest in radio as a response intervention has been so low. This review has identified several gaps of knowledge. The use and health effects of music from a recovery perspective; an impact analysis of radio for specific groups of affected people, e.g., vulnerable groups; and the distribution of radios for listeners are some suggestions for future studies. In order to add knowledge to these gaps, actors using radio should also be encouraged to collect data during their operations and conduct structured evaluations in order to improve best practice and build further evidence. 

## 5. Conclusions

Humanitarian radio has been used to provide health-related information, advice and psychosocial support in natural disasters, thereby contributing to the health and wellbeing of the affected populations from physical, psychosocial and community perspectives. Radio has the potential to reach large populations in severely damaged areas and could, therefore, be a useful and powerful health response intervention after natural disasters. As such, health professionals; crises communication experts, e.g., radio journalists; and disaster managers should be prepared to use humanitarian radio as part of their response operations in natural disasters. 

## Figures and Tables

**Table 1:** Overview of the database search.

Database	Search Terms	Number of Records
**Medline** * Year: 1998–2018 Language: English	S1: [mass casualty] OR [catastrophe] OR [disaster] OR [disaster response] AND [health]	3044
	S2: [mass casualty] OR [catastrophe] OR [disaster] OR [disaster response] AND radio	334
	S3: [disaster] OR [disaster response] AND [radio] AND [health]	10
	S1+S2	128
**Web of Science** * Year: 1998–2018 Language: English Type of source: Article	S1: [mass casualty] OR [catastrophe] OR [disaster] AND [radio]	978
	S2: S1 AND [health]	87
Total papers found		215
After removing duplicates		172
Manual search of grey literature		11
Manual search of reference lists		0
Total number of sources		183

* Search valid as of 4 March 2019.

**Table 2:** Overview of the papers included in the review.

Author/s (Year)	Disaster Country/Region (Year)	Type of Disaster
International Organization for Migration (IOM) (2010)	Haiti (2010)	Earthquake
Barnett et al. (2018)	Western Africa (2014)	Ebola epidemic
Beaudoin (2008)	United States of America (USA)	Hurricane
Beadoin et al. (2009)	USA	Hurricane
Bedrosian et al. (2016)	Western Africa (2014)	Ebola epidemic
Burger et al. (2013)	USA (2012)	Hurricane
First Response Radio (2019)	Several	Several
Gillespie et al. (2016)	Western Africa (2014)	Ebola epidemic
Hannides et al. (2015)	Nepal (2015) Western Africa (2014)	Earthquake Ebola epidemic
Hugelius et al. (2016a)	The Philippines (2013)	Typhoon
Hugelius et al. (2016b)	The Philippines (2013)	Typhoon
Hugelius et al. (2017)	The Philippines (2013)	Typhoon
Rundblad et al. (2010)	United Kingdom (2007)	Flooding

**Table 3:** Overview of First Response Radio (FRR) disaster response operations from January 2004 to April 2019.

Year	Country	Disaster Location	Type of Disaster	Estimated Number of People in the Disaster Location, *Possibly* Covered by FRR Radio *
2004	Indonesia	Banda Aceh	Tsunami	300,000
2005	Pakistan	Kashmir	Earthquake	5,128,309
2008	India	Bihar	Flooding	50,000
2009	Indonesia	Padang	Earthquake	2,501,798
2009	India	South India	Flooding	4,100,000
2009	The Philippines	Pangasinan	Flooding	N/A
2010	India	Jammu and Kashmir	Flash flooding	N/A
2010	Pakistan	Charsadda	Flooding	950,000
2010	Indonesia	Mt. Merapi	Volcano	137,140
2012	India	Assam	Flooding	2,200,000
2013	India	Uttarakhand	Flash flooding	504,000
2013	India	Orissa/Phalin	Cyclone	13,230,000
2013	Indonesia	Ache	Earthquake	55,935
2013	The Philippines	Tacloban	Cyclone	250,000
2014	Indonesia	Mt. Sinabung	Volcano	32,000
2014	Indonesia	Mt. Kelud	Volcano	83,158
2014	Indonesia	Mt. Sangeang	Volcano	29,727
2014	The Philippines	East Samar	Cyclone	113,878
2015	Nepal	Rasuwa	Earthquake	2,800,000
2015	The Philippines	Casiguran	Cyclone	N/A
2015	Pakistan	N/A	Earthquake	502,590
2016	The Philippines	North Luzon	Cyclone	143,029
2016	Indonesia	Aceh	Earthquake	86,018
2017	Indonesia	East Java	Landslide	N/A
2017	India	Bihar	Flooding	100,000
2017	Indonesia	Mt. Agung	Volcano	150,000
2018	The Philippines	Mt. Mayon	Volcano	89,110
2018	India	Kerala	Flooding	5,400,000
2018	Indonesia	Lombok	Earthquake	516,927
2018	Indonesia	Palu	Earthquake/Tsunami	210,894
2018	Indonesia	Banten	Tsunami	14,700
2019	Mozambique	Beira	Cyclone	300,000

* The exact number of people covered by FRR radio has not been reported. The numbers presented are the reported number of affected people in the locations where the radio broadcasts were operative.

## References

[1] Centre for Research on the Epidemiology of Disasters (CRED), Institute Health and Society, Université Catholique de Louvain (2018). Natural Disasters 2017.

[2] Barbour V. Earthquakes, Cyclones, Tsunamis, Floods and Volcanoes—Assessing the Human Impact of Each. https://blogs.plos.org/speakingofmedicine/2013/04/19/earthquakes-cyclones-tsunamis-floods-and-volcanoes-assessing-the-human-impact-of-each/.

[3] Pan American Health Organization/World Health Organization (2000). Natural Disasters. Protecting the Public´s Health.

[4] Bartels S.A., van Royen M.J. (2011). Medical complication associated with earthquakes. Lancet.

[5] Sundnes K.N., Birnbaum M.L. (2003). Health Disaster Management. Guidelines for Evaluation and Research in the Utstein style. Conceptual framework of Disasters. Prehospital Disaster Med..

[6] Arnberg F., Gudmundsdottir R., Butwicka A., Fang F., Lichtenstein P., Hultman C.M., Valdimarsdottir U.A. (2015). Psychiatric disorders and suicide attempts in Swedish survivors of the 2004 Southeast Asia tsunami: A 5 year matched cohort study. Lancet Psychiatry.

[7] Bonanno G.A., Brewin C.R., Kaniasty K., La Greca A.M. (2010). Weighing the Costs of Disaster: Consequences, Risks, and Resilience in Individuals, Families, and Communities. Psychol. Sci. Public Interest.

[8] Lock S., Rubin G.J., Murray V., Rogers M.B., Amlôt R., Williams R. (2012). Secondary stressors and extreme events and disasters: A systematic review of primary research from 2010–2011. PLoS Curr..

[9] Waelveerakup W. (2014). The quality of life of flood survivors in Thailand, Nakthon Pathom Rajabhat University. Australas. Emerg. Nurs. J..

[10] Bradley D.T., McFarland M., Clarke M. (2014). The Effectiveness of Disaster Risk Communication: A Systematic Review of Intervention Studies. PLoS Curr. Disasters.

[11] CHS Alliance Core Humanitarian Standard on Quality and Accountability. Core Humanitarian Standard 2019. https://corehumanitarianstandard.org/the-standard.

[12] Longstaff P.H., Yang S. (2008). Communication management and trust: Their role in building resilience to “surprises” such as natural disasters, pandemic flu, and terrorism. Ecol. Soc..

[13] Rogers M.B., Amlot R., Rubin G.J., Wessley S., Krieger K. (2007). Mediating the social and psychological impacts of terrorisms attacks: The role of perception and risk communication. Int. Rev. Psychiatry.

[14] International Federation of Red Cross and Red Crescent Societies (2013). World Disaster Report 2013. Focus on Technology and the Future of Humanitarian Action.

[15] Romo-Murphy E., James R., Adams M. (2011). Facilitating disaster preparedness trough local radio broadcasting. Disasters.

[16] Whittemore R., Knafl K. (2005). The integrative review: Updated methodology. J. Adv. Nurs..

[17] Communication with Disaster Affected Communities (CDAC). http://www.cdacnetwork.org/members/.

[18] International Organization of Migration (IOM) Haiti: The Role of Media in the Reconstruction. https://reliefweb.int/report/haiti/haiti-role-media-reconstruction.

[19] Barnett S., van Dijk J., Swaray A., Amara T., Young P. (2018). Redesigning an education project for child friendly radio: A multisectoral collaboration to promote children’s health, education, and human rights after a humanitarian crisis in Sierra Leone. BMJ.

[20] Burger J., Gochfeldt M., Jeitner C., Pittfield T., Donio M. (2013). Trusted Information Sources Used During and After Superstorm Sandy: TV and Radio were Used More Often than Social Media. J. Toxicol. Environ. Health.

[21] Gillespie A.M., Obregon R., El Asawi R., Richey C., Manoncourt E., Joshi K., Naqvi S., Pouye A., Safi N., Chitnis K. (2016). Social Mobilization and Community Engagement Central to the Ebola Response in West Africa: Lessons for Future Public Health Emergencies. Glob. Health Sci. Pract..

[22] Hugelius K., Gifford M., Ortenwall P., Adolfsson A. (2016). Disaster Radio for Communication of Vital messages and Health-related Information; analysis from the Haiyan typhoon, The Philippines. Disaster Med. Public Health Prep..

[23] First Response Radio (2019). Summery of Operations Year 2004 to 2019.

[24] Hannides T. (2015). Humanitarian Broadcasting in Emergencies. A Synthesis of Evaluation Findings.

[25] Hugelius K. (2017). Disaster Response for Recovery. Survivors’ Experiences and the Use of Disaster Radio to Promote Health after Natural Disasters. Ph.D. Thesis.

[26] Beaudoin C.E. (2008). Assessment of a media campaign and related crisis help line following Hurricane Katrina. Public Health Rep..

[27] Beaudoin C.E. (2009). Evaluating a media campaign that targeted PTSD after Hurricane Katrina. Health Commun..

[28] Rundblad G., Knapton O., Hunter P. (2010). Communication, perceptions and behavior during a natural disaster involving a ‘Do Not Drink’ and subsequent ‘Boil Water’ notice: A postal questionnaire study. BMC Public Health.

[29] Bedrosian S.R., Young C.E., Smith L.A., Manning C., Pecha L., Telfer J.L., Gaines-McCollom M., Daniel K.L. (2016). Lessons of risk communication and health promotion—West Africa and United States. Center for Disease Control and Prevention (CDC). MMWR.

[30] Hugelius K., Gifford M., Örtenwall P., Adolfsson A. (2017). Health among disaster survivors and health professionals after the Haiyan Typhoon: A self-selected Internet-based web survey. Int. J. Emerg. Med..

[31] Hugelius K., Gifford M., Ortenwall P., Adolfsson A. (2016). “To silence the deafening silence”; Experiences of the impact of disaster radio for survivor’s wellbeing after a natural disaster. J. Int. Emerg. Nurs..

[32] Murray J., Head R., Sarrassat S., Hollowell J., Remes P., Lavoie M., Borghi J., Kasteng F., Meda N., Badolo H. (2018). Modelling the effect of a mass radio campaign on child mortality using facility utilization data and the Lives Saved Tool (LiST): Findings from a cluster randomized trial in Burkina Faso. BMJ Glob. Health.

[33] Stallings R.A. (2007). Methodological issues. Handbook of Disaster Research.

[34] Wietsse A.T., Barui C., Galappatti A., Silove D., Betancourt T.S., Souza R., Golaz A.O.M. (2011). Mental health and psychosocial support in humanitarian settings; linking practice and research. Lancet.

[35] Inter-Agency Standing Committee (2013). IACS Guidelines on Mental Health and Psychosocial Support in Emergency Settings.

[36] Hobfoll S.E., Watson P., Bell C.C., Bryant R.A., Brymer M.J., Friedman M.J., Friedman M., Gersons B.P.R., de Long T.V.M., Layne M. (2007). Five Essential Elements of Immediate and Mid-Term Mass Trauma Intervention: Empirical Evidence. Psychiatry.

[37] Lazarus R.S. (2006). Emotions and Interpersonal Relationships; Toward a Person-Centered Conceptualization of Emotions and Coping. J. Personal..

[38] Norris F.H., Elrod C.L., Norris F.H., Galea S., Friedman M.J., Watson P.J. (2006). Psychosocial consequences of disaster. A review of past research. Methods for Disaster Mental Health Research.

[39] Arnberg F., Hultman C.M., Michel M., Lundin T. (2012). Social Support Moderates Posttraumatic Stress and General Distress after Disaster. J. Trauma. Stress.

[40] Southwick S.M., Sippel L., Krystal J., Charney D.S., Mayes L., Pietrzak R.H. (2016). Why are some individuals more resilient than other: The role of social support. World Psychiatry.

[41] Sacks O. (2006). The power of music. Brain.

[42] Norris F.H., Stevens P.S., Pfefferbaum B., Wyche K.F., Pfefferbaum R.L. (2008). Community Resilience as a Metaphor, Theory, Set of Capacities and Strategy for Disaster Readiness. Am. J. Community Psychol..

[43] Rasmussen J., Ihlen Ø. (2017). Risk, Crisis, and Social Media: A systematic review of seven years’ research. Nord. Rev..

[44] International Federation of the Red Cross and Red Crescent Societies (IFRC) (2011). Beneficiary Communication and Accountability: A Responsibility, Not a Choice. Lessons Learned and Recommendations.

[45] Paez A. (2017). Grey literature: An important resource in systematic reviews. J. Evid.-Based Med..

[46] Adams J., Hillier-Brown F.C., Moore H.J., Lake A.A., Araujo-Soares V., White M., Summerbell C. (2016). Searching and synthesizing ‘grey literature’ and ‘grey information’ in public health: Critical reflections on three case studies. Syst. Rev..

[47] Pautasso M. (2013). Ten simple rules for writing a literature review. PLoS Comput. Biol..

